# Supplementary material for: Altered Network Function in Hippocampus After Sub-Chronic Activation of Cannabinoid Receptors in Early Adolescence
Source: Int J Mol Sci. 2025 Dec 18;26(24):12182. doi: 10.3390/ijms262412182 (PMC12734249; doi:10.3390/ijms262412182)
Supplement: Supplementary file 1 [file ijms-26-12182-s001.zip › ijms-3917580-supplementary.pdf]

Supplementary Materials

Altered Network Function in Hippocampus After Sub-Chronic Activation of Cannabinoid Receptors in Early Adolescence

Johanna Rehn [1], Lucas Admeus [2] and Bernat Kocsis [1,*]

1 Department Psychiatry, Beth Israel Deaconess Medical Center, Harvard Medical School, Boston, MA 02115, USA; johannapersonrehn@gmail.com
2 Sahlgrenska Academy, University of Goteborg, 41390 Gothenburg, Sweden; lucasadmeus@gmail.com
* Correspondence: bkocsis@hms.harvard.edu

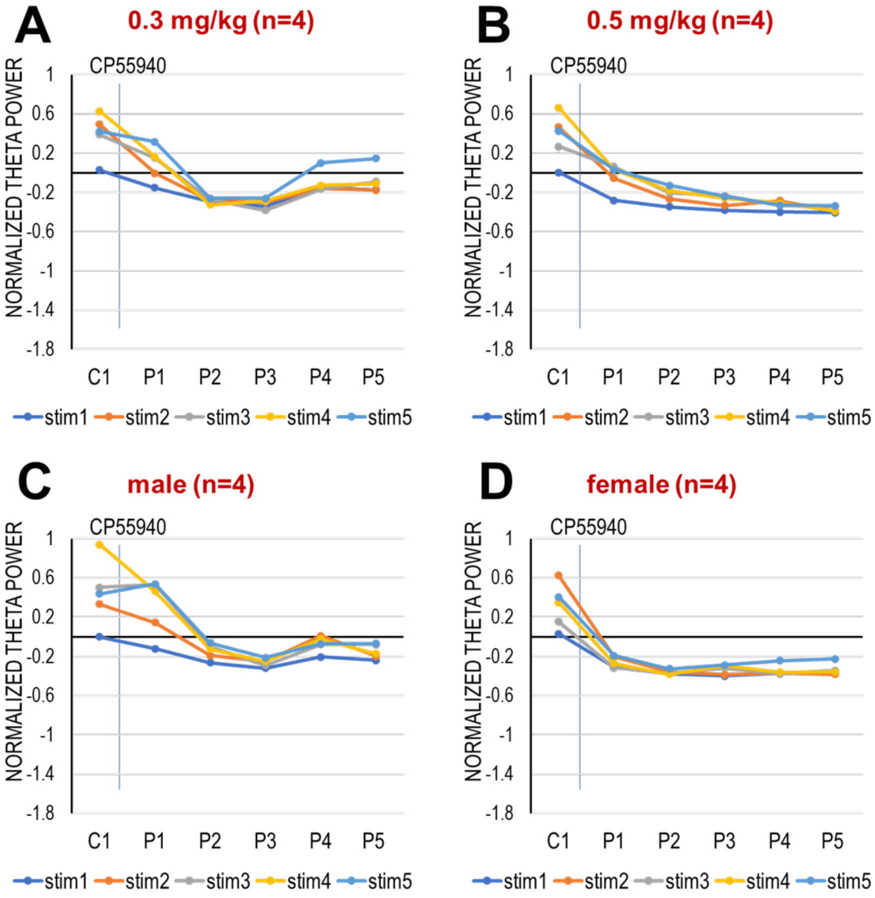

Figure S1. Effect of the testing dose (**A-B**) and sex (**C-D**) on the effect of CB1R activation in adult rats with no prior CB exposure. **A-B.** Effect of CP-55940 injected in different doses (0.3 and 0.5 mg/kg, **A** and **B**, respectively). **C-D.** Effect of CP-55940 injection in adult male (**C**) and female rats (**D**). Note slight differences of theta suppression only expressed in delayed onset (P4-P5) and less stable, shorter reaction (P2-P3) following the lower CP-55940 dose and in male rats.

Table S1. Individual experiments numbered/listed in the order of the date of recording performed under urethane anesthesia in adulthood.

Rat Number	Sex		Adolescent Treatment			Experiment in Adulthood	
			**Timing (PND)**	**Compound**		**at Age (PND)**	**CP-55940 Dose**
1	m		P42-46	vehicle		P72	0.3 mg/kg
2	m		P42-46	CP-55940		P73	0.3 mg/kg
3	f		P42-46	CP-55940		P74	0.3 mg/kg
4	f		P42-46	vehicle		P75	0.3 mg/kg
5	m		P42-46	CP-55940		P76	0.3 mg/kg
6	m		P32-36	vehicle		P70	0.3 mg/kg
7	m		P32-36	CP-55940		P71	0.3 mg/kg
8	f		P32-36	CP-55940		P72	0.3 mg/kg
9	f		P32-36	vehicle		P73	0.3 mg/kg
10	m		P32-36	CP-55940		P74	0.3 mg/kg
11	m		none				vehicle
12	m		none				vehicle
13	f		P42-46	vehicle		P88	0.5 mg/kg
14	f		P42-46	CP-55940		P89	0.5 mg/kg
15	m		P42-46	CP-55940		P90	0.5 mg/kg
16	m		P42-46	vehicle		P91	0.5 mg/kg
17	f		P32-36	vehicle		P85	0.5 mg/kg
18	f		P32-36	CP-55940		P86	0.5 mg/kg
19	m		P32-36	CP-55940		P87	0.5 mg/kg
20	m		P32-36	vehicle		P88	0.5 mg/kg
21	f		P32-36	CP-55940		P111	none
22	f		P42-46	CP-55940		P112	0.5 mg/kg
N=19*	m: n=10<br>f: n=9		Early: n=9<br>Late: n=10	CP-55940: n=11<br>Vehicle: n=9			0.3mg/kg: n=10<br>0.5mg/kg: n=9

* 19 experiments were used for statistical analysis. Rat#21 died at surgery, rats#11-12 were only used to verify experimental conditions, set-ups, to produce result shown in control animals in numerous prior studies (see e.g. [1-4]).

References

1 Hajos, M., Siok, C. J., Hoffmann, W. E., Li, S. & Kocsis, B. Modulation of hippocampal theta oscillation by histamine H3 receptors. *J Pharmacol Exp Ther* **324**, 391-398 (2008). https://doi.org/10.1124/jpet.107.130070

2 Sorman, E., Wang, D., Hajos, M. & Kocsis, B. Control of hippocampal theta rhythm by serotonin: role of 5-HT2c receptors. *Neuropharmacology* **61**, 489-494 (2011). https://doi.org/10.1016/j.neuropharm.2011.01.029

3 Mofleh, R. & Kocsis, B. Respiratory coupling between prefrontal cortex and hippocampus of rats anaesthetized with urethane in theta and non-theta states. *Eur J Neurosci* **54**, 5507-5517 (2021). https://doi.org/10.1111/ejn.15384

4 Thorn, C. W., Kafetzopoulos, V. & Kocsis, B. Differential Effect of Dopamine D4 Receptor Activation on Low-Frequency Oscillations in the Prefrontal Cortex and Hippocampus May Bias the Bidirectional Prefrontal-Hippocampal Coupling. *Int J Mol Sci* **23** (2022). https://doi.org/10.3390/ijms231911705
